# Lifelong exercise training promotes the remodelling of the immune system and prostate signalome in a rat model of prostate carcinogenesis

**DOI:** 10.1007/s11357-023-00806-5

**Published:** 2023-05-12

**Authors:** Elisabete Nascimento-Gonçalves, Fernanda Seixas, Carlos Palmeira, Gabriela Martins, Carolina Fonseca, José Alberto Duarte, Ana I. Faustino-Rocha, Bruno Colaço, Maria João Pires, Maria João Neuparth, Daniel Moreira-Gonçalves, Margarida Fardilha, Magda C. Henriques, Daniela Patrício, Steven Pelech, Rita Ferreira, Paula A. Oliveira

**Affiliations:** 1 Centre for the Research and Technology of Agro-Environmental and Biological Sciences (CITAB), University of Trás-Os-Montes and Alto Douro (UTAD), 5000-801 Vila Real, Portugal; 2 Institute for Innovation, Capacity Building and Sustainability of Agri-Food Production (Inov4Agro), UTAD, 5000-801 Vila Real, Portugal; 3 LAQV-REQUIMTE, Department of Chemistry, University of Aveiro (UA), 3810-193 Aveiro, Portugal; 4 Animal and Veterinary Research Centre (CECAV), Associate Laboratory for Animal and Veterinary Science - AL4AnimalS, UTAD, 5000-801 Vila Real, Portugal; 5 Department of Veterinary Sciences, University of Trás-Os-Montes and Alto Douro, 5000-801 Vila Real, Portugal; 6 Clinical Pathology Department, Portuguese Institute of Oncology of Porto, 4200-072 Porto, Portugal; 7 Experimental Pathology and Therapeutics Group, Portuguese Institute of Oncology, 4200-072 Porto, Portugal; 8 School of Health Science Fernando Pessoa and FP-i3iD, 4200-253 Porto, Portugal; 9 Clinical Pathology Department, Portuguese Institute of Oncology of Porto, 4200-072 Porto, Portugal; 10 Experimental Pathology and Therapeutics Group, Portuguese Institute of Oncology, 4200-072 Porto, Portugal; 11 CIAFEL, Research Centre in Physical Activity, Health and Leisure, Faculty of Sport, University of Porto, 4200-450 Porto, Portugal; 12 Department of Zootechnics, School of Sciences and Technology, University of Évora, 7004-516 Évora, Portugal; 13 Comprehensive Health Research Centre, 7004-516 Évora, Portugal; 14 Animal and Veterinary Research Centre (CECAV), Associate Laboratory for Animal and Veterinary Science – AL4AnimalS, UTAD, 5000-801 Vila Real, Portugal; 15 Department of Zootechnics, University of Trás-Os-Montes and Alto Douro, 5000-801 Vila Real, Portugal; 16 Research Center in Physical Activity, Health and Leisure (CIAFEL)-Faculty of Sports-University of Porto (FADEUP), Portugal and Laboratory for Integrative and Translational Research in Population Health (ITR), Porto, Portugal; 17 TOXRUN – Toxicology Research Unit, University Institute of Health Sciences, CESPU, CRL, 4585-116 Gandra, Portugal; 18 Department of Medical Sciences, iBiMED – Institute of Biomedicine, University of Aveiro, 3810-193 Aveiro, Portugal; 19 Department of Medicine, University of British Columbia, Vancouver, B.C Canada; 20 Kinexus Bioinformatics Corporation, Suite 1 – 8755 Ash Street, Vancouver, BC V6P 6T3 Canada; 21 LAQV-REQUIMTE, Department of Chemistry, University of Aveiro, 3810-193 Aveiro, Portugal; 22 Clinical Academic Center of Trás-Os-Montes and Alto Douro, University of Trás-Os-Montes and Alto Douro, 5000-801 Vila Real, Portugal; 23 University of Trás-os-Montes and Alto Douro, Quinta dos Prados, 5001-801 Vila Real, Portugal

**Keywords:** Animal model, Cancer, Exercise training, Immune system, Prostate

## Abstract

This work aimed to understand how lifelong exercise training promotes the remodelling of the immune system and prostate signalome in a rat model of PCa. Fifty-five male Wistar rats were divided into four groups: control sedentary, control exercised, induced PCa sedentary and induced PCa exercised. Exercised animals were trained in a treadmill for 53 weeks. Pca induction consisted on the sequential administration of flutamide, N-methyl-N-nitrosourea and testosterone propionate implants. Serum concentrations of C-reactive protein (CRP) and tumor necrosis factor (TNF)-like weak inducer of apoptosis (TWEAK) were not different among groups. Peripheral levels of γδ T cells were higher in Pca exercised group than in the PCa sedentary group (*p* < 0.05). Exercise training also induced Oestrogen Receptor (ESR1) upregulation and Mitogen-activated Protein Kinase 13 (MAPK13) downregulation, changed the content of the phosphorylated (at Ser-104) form of this receptor (coded by the gene ESR1) and seemed to increase Erα phosphorylation and activity in exercised PCa rats when compared with sedentary PCa rats. Our data highlight the exercise-induced remodelling of peripheral lymphocyte subpopulations and lymphocyte infiltration in prostate tissue. Moreover, exercise training promotes the remodelling prostate signalome in this rat model of prostate carcinogenesis.

**Supplementary Information:**

The online version contains supplementary material available at 10.1007/s11357-023-00806-5.

## Introduction

Prostate cancer (PCa) is still a major medical, social and economic concern due to the increasing prevalence and unsatisfactory treatment outcomes [1, 2]. Some Pca grow slowly over many years and are confined to the prostate gland and might not need treatment. However, other types are more aggressive and can spread quickly [3]. Thus, it is necessary to reinforce the investment in the scientific research to reverse these data. Many research approaches have been tested, with some of them addressing the interference of exercise in the development and progression of Pca. Study the interplay between PCa and physical exercise is of paramount importance not only to understand the role of exercise in the triggers and mechanisms involved in the carcinogenesis, but also to implement training protocols adjusted to each PCa patient. Even though have been published several studies concerning to the benefits of exercise in PCa setting, the biological mechanisms by which exercise may prevent PCa development and progression remain unknown [4–6]. It is presumed that exercise delays PCa progression through the modulation of circulating factors, hormone receptor adaptation, reduced systemic inflammation and improved immune function [5].

Moreover, epidemiological and animal studies suggest that physical activity influence positively immune cell population and their functions [7, 8]. During physical exercise antipathogen activity of tissue macrophages increase in parallel with an enhanced recirculation of immunoglobulins [8]. It is also described an increase in the levels of anti-inflammatory cytokines, neutrophils, natural killer (NK) cells, cytotoxic T cells, and immature B cells in tissues, all of which play critical activities in immune activity [7]. These events modulated by exercise together contribute to enhance immune defense against cancer.

The study of exercise training, particularly lifelong exercise, in the cancer setting is a challenge which explains the lack of lifelong exercise protocols to better understand its influence on cancer development. When compared with other animals, rodents offer the advantage of short lifespan, allowing to study the preventive effects of lifelong exercise in cancer development. Furthermore, male dorsolateral prostate rat and man prostate shares features regarding development, function and susceptibility to chemical carcinogens [9], which led to the recognition of male rats as a suitable model for study prostate carcinogenesis [10, 11].

Taking all these facts in consideration, the present work aimed to understand the effects of lifelong moderate exercise in PCa development in an animal model. Thus, we investigated the modulation of PCa development by physical exercise, addressing the potential contribution of the immune system to this interplay through the flow cytometry study of peripheral immune cell populations and the immunohistochemical analysis of cell proliferation, androgen receptor (AR), estrogen receptor (ER) and vascular endothelial growth factor (VEGF) expression. Moreover, we searched for the signalling pathways modulated by exercise in dorsolateral prostate tissue using a microarray-based approach. We observed that physical exercise modulated the immune system and prostate signalome, which may contribute to improve exercise prescription for the prevention and treatment of PCa, supporting the vision that “Exercise is Medicine” as previously established in 2007 by the American College of Sports Medicine [12]

## Material and methods

### Animals

All the animal experiments were approved by the Institutional Animals Ethics Committee and by Portuguese national authorities (*Direção Geral de Alimentação e Veterinária*, approval number 021326). Fifty-five 4-week-old male Wistar Unilever rats (*Rattus norvegicus*) were purchased from Charles River Laboratories (France), acclimatized for one week prior to the start of the experiment and were kept at animal facilities of University of Trás-os-Montes and Alto Douro, at controlled environmental conditions: temperature (22 ± 2ºC) and humidity (50 ± 10%). The 12 h light (20.00–08.00 h) and dark cycle were maintained throughout the study. Animals allowed to access food and water ad libitum (Mucedola 4RF21®, Milan, Italy).

### Implementation of exercise training and induction of prostate tumorigenesis

Animals were randomly divided into four groups as follow: control sedentary group – SED + CONT (*n* = 10), control exercised group – EX + CONT (*n* = 10), induced sedentary group – SED + PCa (*n* = 15), and induced exercised group – EX + PCa (*n* = 20). The animals from exercised groups started the exercise training program in a treadmill (Treadmill Control LE 8710, Harvard Apparatus®, USA), at the age of eight weeks and this program was maintained for 53 weeks (5 days per week). The exercise training program started with 30 min *per* day during the first week (habituation period) and was then increased to 60 min per day for the duration of the regimen. The speed of the treadmill was set to 70% of the maximal speed capacity of the animals with induced PCa and, every 15 days, the speed capacity was re-evaluated to correct exercise intensity, as published by Rodrigues et al. (2007) [13]. In order to submit the sedentary animals to similar conditions of exercised animals, they were placed on a stationary treadmill 15 min per day, 5 days per week, over the exercise protocol. Both exercised and non-exercised groups were also subjected to treadmill acclimatization before the beginning of the exercise protocol. The PCa induction protocol was based in Bosland works [14, 15]: at 12 weeks of age, animals from PCa-induced groups (SED + PCa and EX + PCa) received a subcutaneous administration of the anti-androgenic drug flutamide (50 mg/kg; TCI Chemicals®, Portland, OR, USA) for 21 consecutive days. Twenty-four hours after the last flutamide administration, testosterone propionate (TCI Chemicals®, Portland, OR, USA) was dissolved in corn oil and subcutaneously administered to the animals at a dose of 100 mg/kg. Forty-eight hours later, they were intraperitoneally injected with the carcinogen agent MNU (Isopac®, Sigma Chemical Co., Madrid, Spain) at a dose of 30 mg/kg. Two weeks later, silastic tubes were filled with crystalline testosterone (Sigma® Chemical Co., Madrid, Spain) and subcutaneously implanted in the interscapular region of animals previously anesthetized with ketamine (75 mg/kg, Imalgene® 1000, Merial S.A.S., Lyon, France) and xylazine (10 mg/kg, Rompun® 2%, Bayer Healthcare S.A., Kiel, Germany) until the end of experimental protocol. Animals from control groups (SED + CONT and EX + CONT) were handled in the same way, administered with normal saline solution and silastic tubes without testosterone were implanted in the interscapular region, under anaesthesia. Flutamide induces prostate atrophy and prepare the prostate for a maximal cell proliferation response to the subsequent testosterone propionate treatment. The subsequent administration of testosterone propionate induces accelerated multiplication of prostatic cells. Thus, at the time of administration of the carcinogen (MNU), forty-eight hours later, the prostate cells are at the peak of their division, and the carcinogenic capacity of this agent is potentiated [15, 16]. Animals from EX experimental groups adapted well to the exercise training protocol. The remaining animals were sacrificed at 61 weeks of age through an intraperitoneal injection of ketamine (75 mg/kg, Imalgene® 1000, Merial S.A.S., Lyon, France) and xylazine (10 mg/kg, Rompun® 2%, Bayer Healthcare S.A., Kiel, Germany), followed by exsanguination by cardiac puncture. An aliquot of blood was collected for a dry tube (for serum separation) and other aliquot was collected for a tube with an EDTA salt as an anticoagulant (for flow cytometry). Tibias of all animals were measured using a calliper. The anterior prostate lobes and seminal vesicle were individualized and weighed separately. The dorsolateral prostate surrounding prostatic urethra were weight as a block. A portion of identical size was removed from the same regions of dorsolateral prostate of each animal and was used to analyse antibody microarray and another portion was used to histopathological and immunohistochemical analysis.

### Histopathological, morphological and immunohistochemical analysis of prostate sections

After fixation in 10% neutral buffered formalin, dorsolateral prostate was embedded in paraffin. Tissues sections of 3 µm were processed and stained with Haematoxylin and Eosin (H&E) for observation under optical microscopy and histological classification. All preneoplastic and neoplastic lesions were recorded according to Bosland (1998) [17]. In results analysis was calculated the mean number of dorsolateral prostate lesions *per* group.

The immunohistochemical analysis was performed using a polymeric labelling methodology (NovoLink Polymer Detection System®, Leica Biosystems, Newcastle, UK) following the manufacturer’s instructions. The immunostaining evaluation of estrogen receptor α (ERα, 6F11, Novocastra®, Newcastle), androgen receptor (AR, PG21, MerckMilipore®, Darmstadt, Germany) and vascular endothelial growth factor (VEGF, clone JH121, Merck Millipore®, Darmstadt, Germany) were based in previously published work [18–21]. We also performed negative control for all reaction to allow better interpretation of specific staining at the antigen site. Phosphate buffered saline (PBS) as the same concentration as the primary antibody was used as a negative control reagent. Briefly, the labelling was assessed in dorsolateral prostate cells in high power magnification (HPM) semi-quantitatively as: ≤ 25% of the cells stained or > 25% of the cells stained for ER α; and ≤ 75% of the cells positive or > 75% of cells positive for AR and VEGF. The Ki-67 (clone MIB-1, Dako, Glostrup, Denmark) expression was also evaluated in a semi-quantitative way as low Ki-67 (positivity 10% of prostate cells). Evaluation of CD4 + (EPR6855, Abcam®, Cambridge, UK), CD8 + (OX-8, Abcam®, Cambridge, UK), CD68 (ab125212, Abcam®, Cambridge, UK), CD163 (EPR19518, Abcam®, Cambridge, UK) and FoxP3 (FJK16s, eBioscience, Invitrogen®, Waltham, USA) counting was carried out in three randomly HPM in the stroma and in the prostate epithelium. For negative controls, primary antibodies were replaced by phosphate buffered saline (PBS). The histopathological classification and the immunohistochemical evaluation were performed by an experienced pathologist (Fernanda Seixas).

### Antibody microarray analysis of prostate tissue

The 39 dorsolateral prostate tissue samples (SED + CONT, n = 9; SED + PCa, n = 10; EX + CONT, n = 10; and EX + PCA, n = 10) were thawed on ice and homogenised in ice-cold lysis buffer (20 mM MOPS, 2 mM EGTA, 5 mM EDTA, 50 mM sodium fluoride, 60 mM β-glycerophosphate, 25 mM sodium pyrophosphate, 5 mM sodium orthovanadate, and 1% Triton X-100, supplemented with 1 mM PMSF, 3 mM benzamidine, 5 µM pepstatin A, 10 µM leupeptin and 1 mM DTT). Lysates were centrifuged at 90,000 × *g* for 30 min at 25 °C, according to the protocol provided by the Kinexus Bioinformatics Corporation (Vancouver, Canada). The resulting supernatants were immediately frozen and kept at -70 °C until used. At Kinexus Bioinformatics Corporation, samples were pooled per group and fluorescently labelled. Purified dye-labelled native proteins were incubated on a Kinex™ KAM-1325 Antibody Microarray (Kinexus, Vancouver, Canada) and analysed in duplicate with 1326 antibodies: 875 phosphosite-specific and 451 pan-specific.

The enrichment analysis tool provided by Gene Ontology Consortium was used to assess the biological relevance of the differentially expressed proteins [22, 23]. Protein ANalysis THrough Evolutionary Relationships (PANTHER) (version 16.0, released 28/07/2020) was used to identify PANTHER protein classes, and GO Ontology database (released 09/10/2020) was used to identify biological processes, molecular functions, and cellular components overrepresented in our data when compared to a *Homo Sapiens* (all genes in database) reference list [24, 25]. The significance of the results was set up to *p* value of ≤ 0.05 after applying the Fisher’s Exact with false discovery rate (FDR) multiple test correction. The results were ordered by fold enrichment (F.E.) and non-relevant processes for prostate morphology and/or physiology were excluded from the analysis.

#### Protein–protein interactions between differentially expressed proteins and in silico analysis of relevant pathways

To assess the interactions between the differentially regulated proteins, protein–protein networks were constructed. For that, we used a combination of deep-curated databases that included the International Molecular Exchange Consortium (IMEx) [26] and Human Integrated Protein–Protein Interaction rEference (HIPPIE) databases (version 2.0) [27] that provide a comprehensive network visualization and to construct, merge, and analyse the networks we use the Cytoscape software (version 3.8.2) [28].

The role of differentially regulated proteins found was also investigated through comprehensive searches in literature and databases. The enrichment in signalling pathways was evaluated using data from Kyoto Encyclopedia of Genes and Genomes (KEGG) database [29], through DAVID bioinformatics Resources (version 2021) [30].

### Blood samples analysing

Aliquots of blood samples were allowed to clot and centrifuged at 3000 rpm for 15 min (4 °C). The serum was separated and frozen at -80 °C until use. Testosterone concentration was assessed using an ELISA Kit (582,701; Caymann Chemical®, MI, USA), following the manufacturer’s instructions. The serum concentrations of C-reactive protein (CRP) and tumor necrosis factor (TNF)-like weak inducer of apoptosis (TWEAK) were determined by immunoblotting as previously described [31].

### Immunophenotyping analysis by flow cytometry

For the flow cytometry analysis, two tubes were prepared for each sample with 100 µL of blood In tube 1 were added the following conjugated monoclonal antibodies (mAbs): cyCD3-BV421 (clone 14A2), CD45-BV510 (clone 30-F11), CD3-FITC (clone 17A2,), CD8a-PerCP-CY (clone 53–6.7), CD4-PE/Cy7 (clone GK1.5) and CD45RA-APC/Cy7 (clone OX-33). In tube 2, the following conjugated monoclonal antibodies were added: CD3-BV421, CD45-BV510, CD161-FITC, CD127-PE, CD8a-PerCP, CD4-PE/Cy7 and CD25 APC (clone PC61). Surface and cytoplasmic antigen labelling were done following standards protocols and manufacturer’s instructions. All the antibodies were provided from BD Biosciences® (Franklin Lakes, NJ, USA). The flow cytometry immunophenotyping was performed using a BD FASC CANTO™ II cytometer (BD Biosciences) and data were analysed with INFINICYT™ software (1.7 version).

### Statistical analysis

Statistical analysis was performed using the SPSS program (Statistical Package for Social Sciences, Chicago, IL, USA) version 25. Results are presented as mean ± standard error (SE) or as median. The normality of the data was checked with the Shapiro–Wilk test. Statistical differences between groups were assessed by two-way analysis of variance (ANOVA) for independent samples, followed by Bonferroni post hoc tests, when the data was normally distributed. In the other cases, the Kruskal–Wallis test was used. Histological and immunohistochemical results were analysed using Chi-square test. The Pearson correlation was used to evaluate the association of prostate weight and final body weight and relative prostate weight and serum testosterone concentration. The *p* ≤ 0.05 values were considered statistically significant.

## Results

### Characterization of animals’ response to prostate carcinogenesis and exercise training

During the experimental protocol four animals died (three animals from EX + PCa group and one animal from PCa + SED group). Data from these animals were not included in the study.

**Fig. 1:**
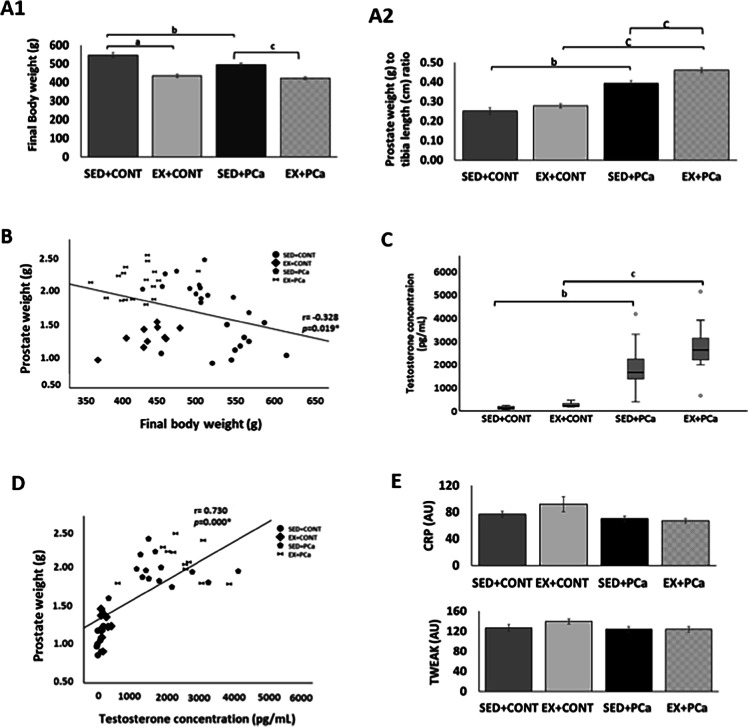
Characterization of animals’ response to prostate carcinogenesis and exercise training. (A1)Rats final body weight (g); (A2) Dorsolateral prostate weight (g) to tibia length (cm) ratio; (B) Correlation between dorsolateral prostate weight (g) and final body weight (g). *Significant correlation at *p* < 0.05; (C) Testosterone concentration (pg/mL); (D) Correlation between serum testosterone concentration (pg/mL) and relative dorsolateral prostate weight (g).* significant correlation at *p* < 0.05; (E) Serum CRP and TWEAK content (arbitrary unit—AU); Mean ± SE. ^a^statistically different from EX + CONT group; ^b^statistically different from SED + PCa group; ^c^statistically different from EX + PCa group. *p* < 0.05

The effect of exercise and PCa interplay on animals’ final body weight and dorsolateral prostate weight can be seen in Fig. 1. Mean body weight was lower in exercised groups (EX + CONT: 434.8 ± 9.4 g and EX + PCa: 421.6 ± 7.6 g) than in sedentary groups (SED + CONT: 546.3 ± 13.5 g and SED + PCa: 494.6 ± 9.5 g; *p* < 0.05; Fig. 1A1). PCa groups had lower mean body weight than respective control groups. Indexing dorsolateral prostate size for tibia length surrogate for body weight was performed to check for biases due to disease-induced body weight changes [32]. Thus, the comparison of prostate weight-to-tibia length ratio among groups showed that exercise training increased prostate weight in control and in PCa groups (*p* < 0.05; Fig. 1A2). A significant negative correlation was found between prostate weight and final body weight (r = -0.328, *p* = 0.019; Fig. 1B).

Circulating testosterone concentration increased in exercised groups when compared with sedentary groups; however, this difference did not reach statistical significance (Fig. 1C). As expected PCa groups (SED + PCa and EX + PCa) had higher serum testosterone concentration, 14% and 10%, than the respective controls groups (SED + CONT and EX + CONT; *p* < 0.05; Fig. 1C), which can be explained by the methodology used to induce the PCa. A significant positive correlation was found between relative prostate weight and serum testosterone concentration (r = 0.730, *p* < 0.0001 Fig. 1D). The circulating levels of the acute phase protein CRP and pro-inflammatory cytokine from TNF family, TWEAK, did not show significant differences among groups (*p* > 0.05; Fig. 1E), suggesting there was no systemic inflammation.

### Immunophenotyping analysis by flow cytometry

**Fig. 2:**
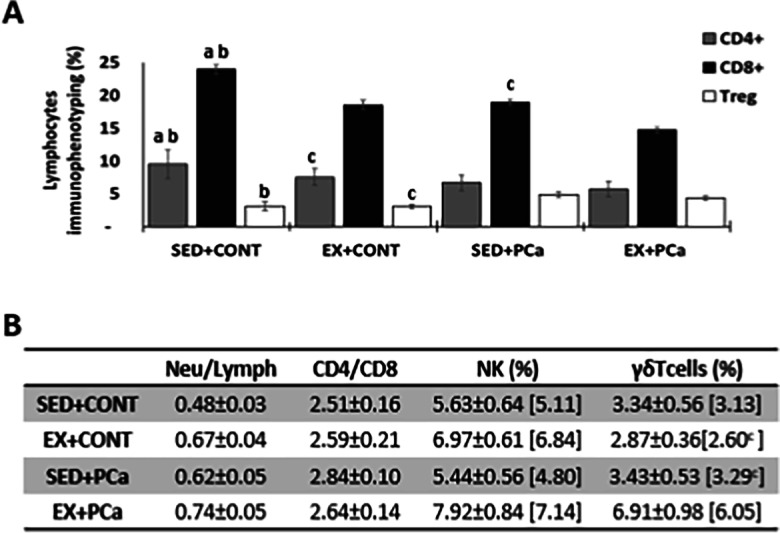
Immunophenotyping analysis by flow cytometry. (A) Percentage of CD4^+^, CD8^+^, Treg cells in the four experimental groups; (B) Neut/Lymph ratio, CD4/CD8 ratio, percentage of natural killer (NK) cells and γδ Tcells (within T-cells) of four experimental groups. Mean ± SE [median]; ^a^statistically different from EX + CONT group; ^b^statistically different from SED + PCa group; ^c^statistically different from EX + PCa group. *p* < 0.05

Results of circulating lymphocytes subpopulations analysed by flow cytometry are presented in Fig. 2A and B. Despite this small decrease in lymphocyte percentage, the proportion of CD4^+^ (T-lymphocytes helper cells) and CD8^+^ (T-lymphocytes cytotoxic cells) cells changed in Ex + PCa group when compared with SED + PCa group, with a lower CD4/CD8 ratio (2.64 vs. 2.84, *p* > 0.05; Fig. 2B). The increase in seric CD8^+^ T-cells compared to CD4^+^ T-cells translates into a greater presence of T-cells with cytotoxic capacity, essential for the immune response against cancer.

Although not statistically significant, a slight decrease in regulatory T cells (Tregs) was observed between the group EX + PCa and the group SED + PCa. Tregs are a specialized subpopulation of T cells involved in tumor development and progression by inhibiting antitumor immunity (4.33 and 4.84, respectively; *p* > 0.05; Fig. 2A). Although the differences were not statistically significant, CD161^+^ (natural killer (NK) cell receptor) exhibited higher levels in exercised groups (EX + CONT and EX + PCa) than in sedentary groups (SED + PCa and SED + PCa, respectively, *p* = 0.06; Fig. 2B). Supplementary Figure 1 show a representative flow cytometry plots of NK cell population comparing one animal from the group induced sedentary (SED + PCa) with one animal from the group induced exercised (EX + PCa).

The T cells (CD3 +) with the CD4-CD8-/ + immunophenotype correspond to γδ T cells, a cytotoxic and antitumour lymphocytes population [33]. Physical exercise increased the percentage of γδ T cells in Ex + PCa group when compared to SED + PCa group as can be seen in Fig. 2B (*p* < 0.05).

### Histological and immunohistochemical analysis of the dorsolateral prostate

**Fig. 3:**
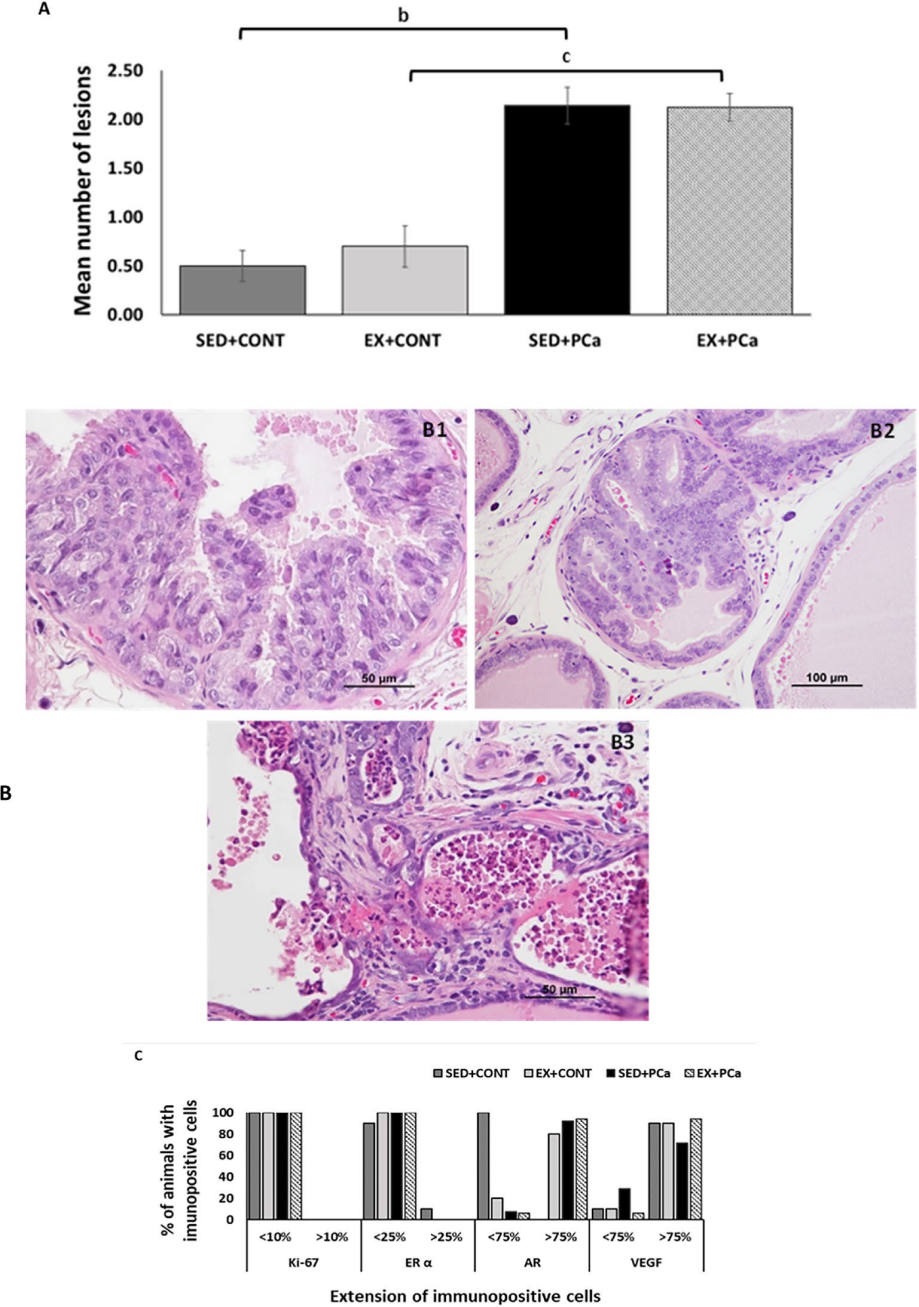

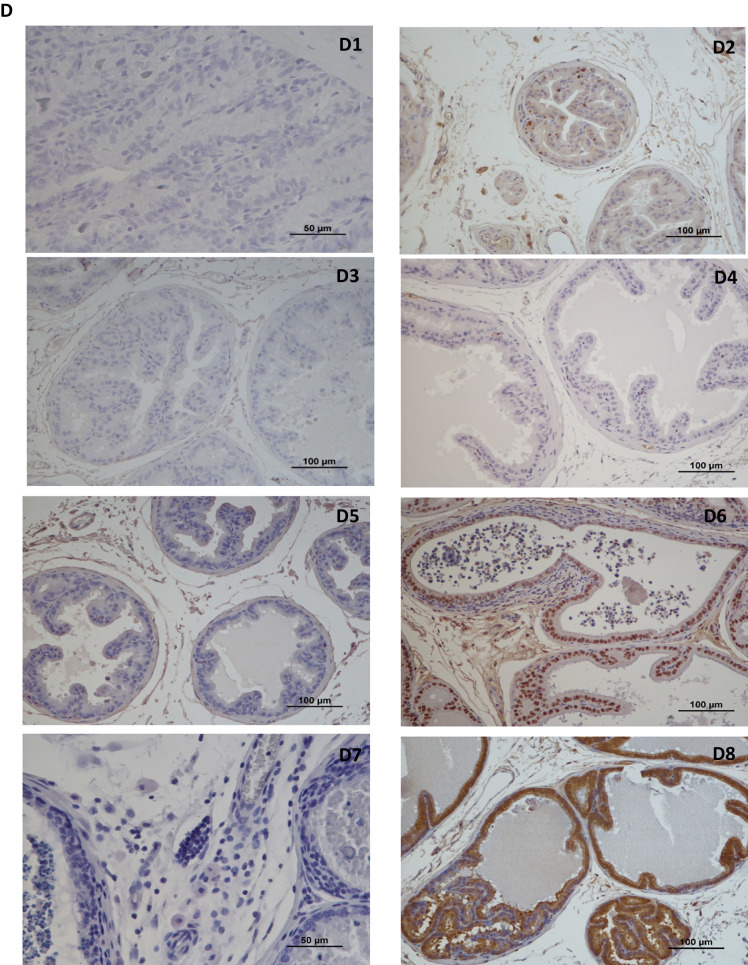
Histological and immunohistochemical analysis of the dorsolateral prostate tissue. (A) Mean number of total dorsolateral prostate lesions in each experimental group; (B) Histopathological lesions observed in dorsolateral prostate tissue. B1 – Dysplasia; B2 – PIN; B3 – Microinvasive carcinoma; (C) % of animals with immunohistochemical expression of Ki-67, estrogen receptor α (ER α), androgen receptor (AR) and vascular endothelial growth factor (VEGF); (D) Immunohistochemical staining D1 – Ki-67 negative control with mitosis (arrow); D2 – Ki-67; D3 – ERα negative control; D4– ERα; D5 – AR negative control; D6 – AR; D7 – VEGF negative control in fibroblasts, macrophages and endothelial cels (arrows); D8 – VEGF.; Mean ± SE. ^b^statistically different from SED + PCa group; ^c^statistically different from EX + PCa group. *p* < 0.05

During necropsy, besides changes in size, no significant macroscopic lesions were observed in the dorsolateral prostate of all animals. However, histological lesions as dysplasia, prostatic intraepithelial neoplasia (PIN) and microinvasive carcinoma were identified in dorsolateral prostate (Fig. 3B).Although CONT groups (SED + CONT and EX + CONT) also developed prostate lesions, the mean number of lesions was lower than in PCa induced groups (SED + PCa and EX + PCa, *p* < 0.05; Fig. 3A).

The tumour proliferation, evaluated by Ki-67 expression, was low (< 10% of the cells were positive, *p* > 0.05; Fig. 3C) and similar among groups. Most of the animals, from all groups, showed low immunopositivity for ERα (less than 25% in all groups), with similar values between sedentary and exercised groups (*p* > 0.05; Fig. 3C). Inversely, all groups showed high immunopositivity for AR. All animals from SED + PCa group had less than 75% AR immunopositive cells in contrast with just 20% of animals from EX + CONT group (*p* < 0.05; Fig. 3C). Physical exercise did not affect AR imunoexpression in PCa groups. All groups showed high VEGF immunopositivity (> 75% of the cells were positive), without differences among groups (*p* > 0.05; Fig. 3C).

### Physical exercise as a modulator of inflammation and fibrosis in dorsolateral prostate

**Fig. 4:**
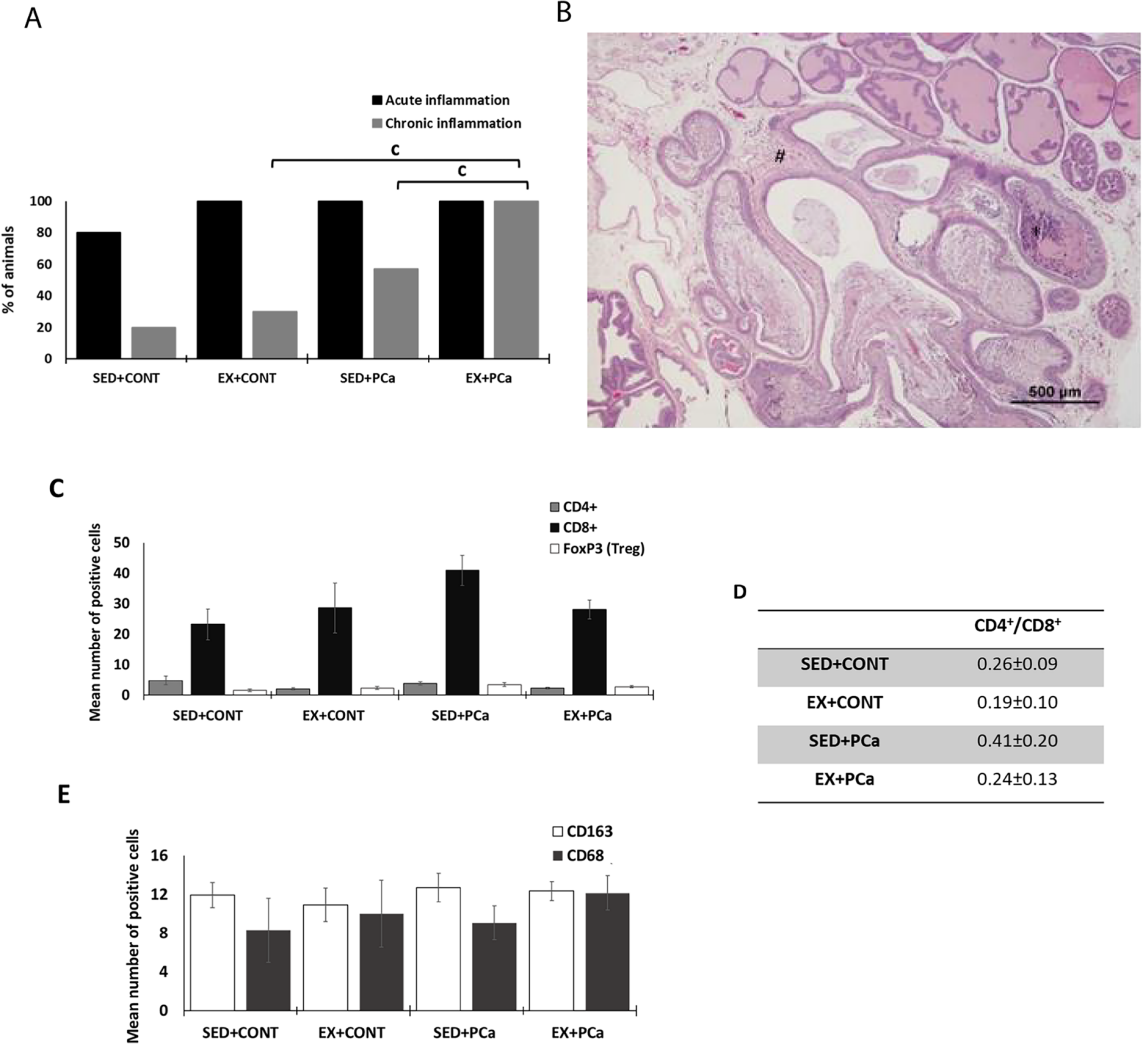

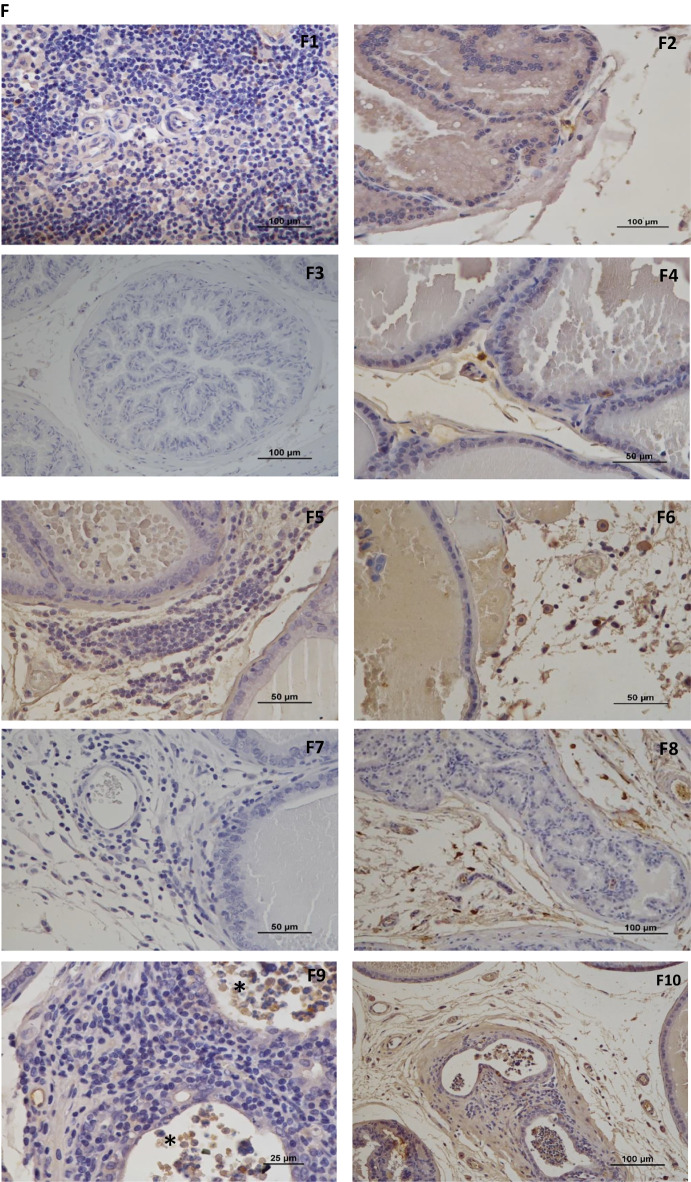
Physical exercise as a modulator of the inflammation and fibrosis in dorsolateral prostate tissue. *(*A) % of animals with acute and chronic inflammation in dorsolateral prostate lobe; (B) Acute Inflammation inside prostate acini (*) and chronic inflammation in the prostate stroma (#) with focal fibrosis. H&E staining; (C) Immunohistochemical detection of CD4^+^, CD8^+^ and FoxP3 (Treg) positive cells in dorsolateral prostate tissue; (D) CD4/CD8 ratio (E) Immunohistochemical detection of CD163 and CD68 positive cells in dorsolateral prostate tissue; (F) Immunohistochemical staining; F1—CD4^+^ negative control, F2- CD4^+^; F3 – CD8^+^ negative control, F4—CD8^+^; F5—FoxP3 negative control; F6 – FoxP3; F7 – CD163 negative control; F8 – CD163; F9—CD68 negative control with adsorption of antibody by necrotic cells (*) and F10 – CD68; Mean ± SE. ^a^statistically different from EX + CONT group; ^b^statistically different from SED + PCa group; ^c^statistically different from EX + PCa group. *p* < 0.05

Focal chronic inflammation with stromal fibrosis and mononuclear cell infiltration were identified in the dorsolateral prostate of all groups and was higher in group EX + PCa than in groups SED + PCa and EX + CONT (*p* < 0.05, Fig. 4A and B).

The lymphocytic infiltrating of CD4^+^, CD8^+^ and FoxP3 + Tcells (Tregs), as well as CD68^+^, considered a pan-macrophages marker and CD163^+^, considered M2 pro-tumoral macrophages, were evaluated in the dorsolateral prostate tissue from all experimental groups (Fig. 4C and D). Although the differences did not reach statistical significance, the seric decrease in CD4^+^/ CD8^+^ ratio in EX + PCa group compared to SED + PCa group (Fig. 4D) suggested a greater presence of cytotoxic T cells (CD8^+^) in EX + PCa group, which have an important role in anti-tumour immunity. Even in a very slight way, there was a decrease of FoxP3^+^ T cells in dorsolateral prostate tissue, *i.e.*, immunosuppressive T cells (Tregs), in EX-PCa group when compared with SED + PCa group (Fig. 4C). On the other hand, an increase in CD68 + expression was observed in EX + PCa group when compared to SED + PCa group (12.14 *vs.* 9.04 positive cells, *p* > 0.05; Fig. 4E). Figure 4F shows representative images of CD4^+^, CD8^+^, FoxP3 + (Treg), CD163^+^ and CD68^+^ immunopositive cells in dorsolateral prostate tissue and respective negative controls.

### Identification of signalling pathways modulated by exercise in PCa

After statistical analysis of the unbiased antibody microarray, 15 proteins were significantly altered in prostate tissue from SED + PCa rats compared to paired tissue from SED + CONT rats, either in terms of basal expression or phosphoacceptor-residue phosphorylation (Table 1). FOXO1 upregulation (+ 115% change from SED + CONT rats) and EIF4G1 (-82% change from SED + CONT rats) were the most prominent changes. Additionally, exercise training also induced alterations in prostate proteome profile (Table 2). In fact, 25 proteins were identified as significantly altered in prostate tissue from EX + PCa rats compared with SED + PCa rats, either in terms of basal expression or phosphoacceptor-residue regulation. The most prominent changes in prostate proteome induce by exercise were estrogen Receptor-alpha (ERα; ESR1) upregulation of stimulatory Ser-104 phosphorylation (+ 1976% change from SED + PCa rats) and Mitogen-activated Protein Kinase 13 (MAPK13; p38δ MAPK) downregulation of stimulatory Thr-180 and Tyr-182 phosphorylation (-80% change from SED + PCa rats).

**Table 1.**
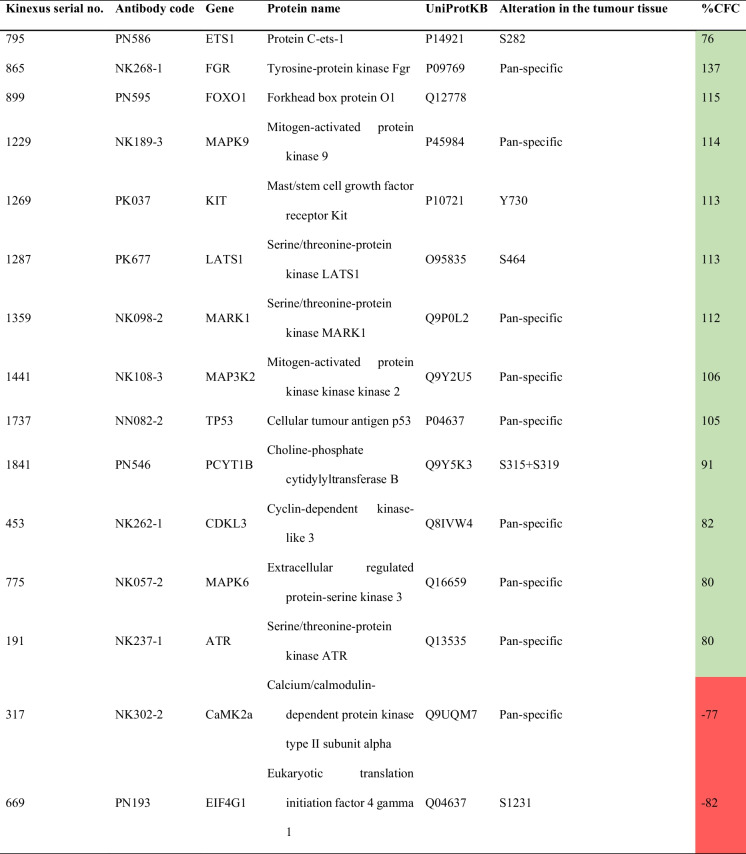
Protein alterations found in the prostate tissue of sedentary PCa rats compared with sedentary CONT rats, using the Kinex^TM^ KAM-1325 Antibody Microarray

Fold change is reflected by the Z-ratio between PCa sedentary rats’ tissue and sedentary control rats’ tissue and the percentage change of the Pca sedentary rats tissue and sedentary control rats tissue is indicated as the percentage from control (%CFC)

**Table 2.**
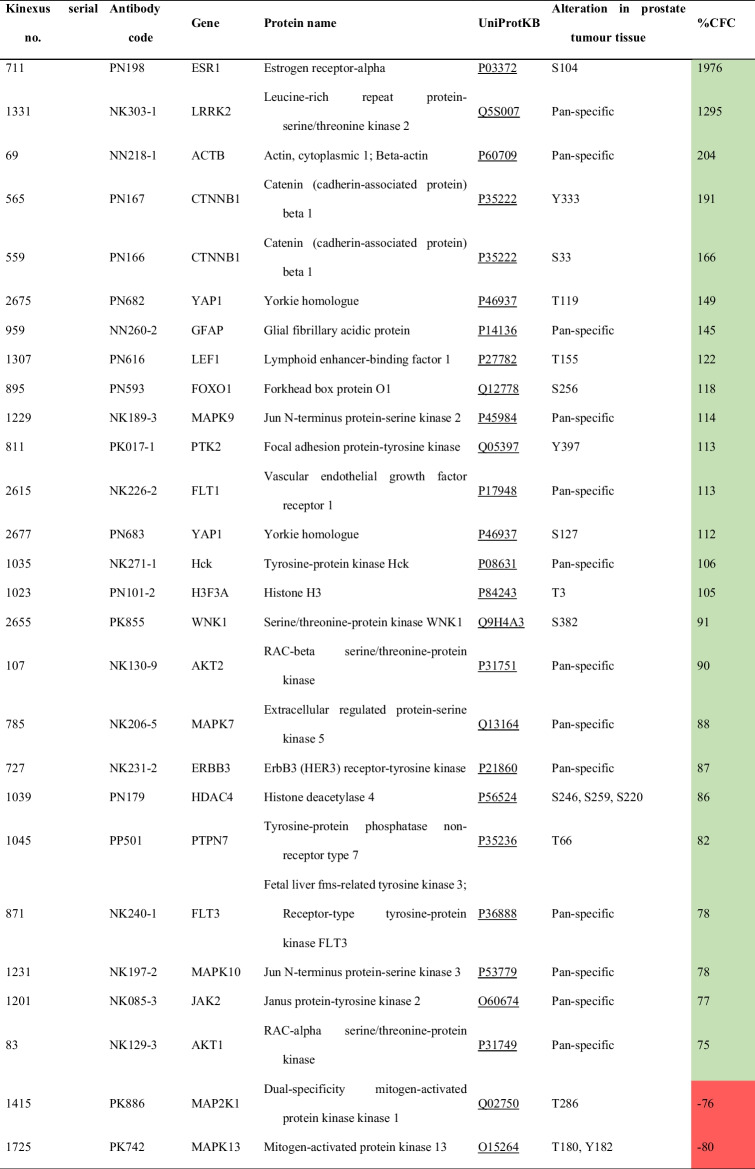
Protein alterations found in the prostate tissue from exercised Pca rats compared with sedentary Pca rats, using the Kinex^TM^ KAM -1325 Antibody Microarray

Fold change is reflected by the Z-ratio between PCa exercised rats’ tissue and PCa sedentary rats’ tissue and the percentage change of the PCa exercised rats’ tissue and PCa sedentary rats’ tissue is indicated as the percentage from control (%CFC)

**Fig. 5:**
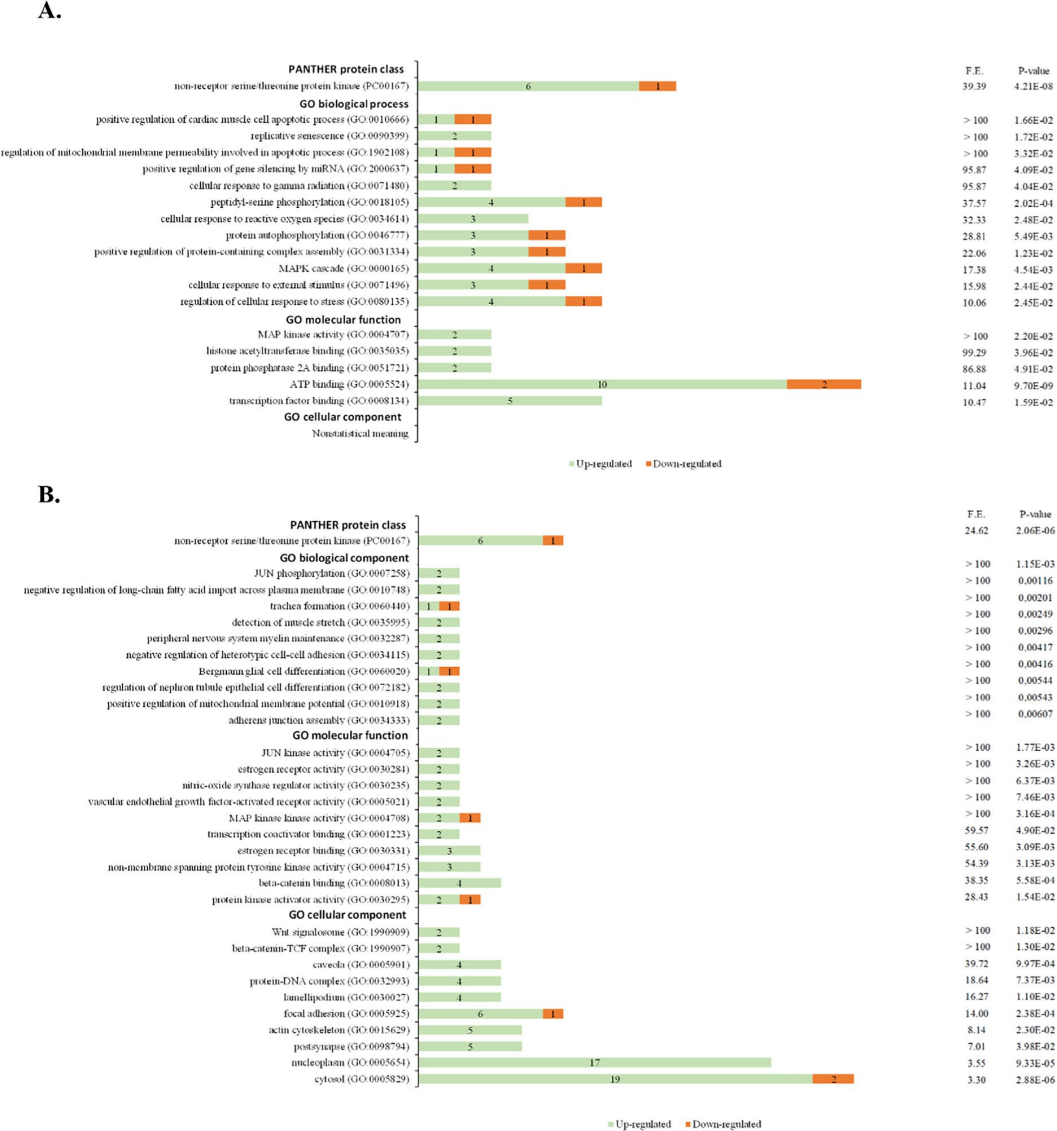
Overrepresented PANTHER protein classes and GO ontology categories of prostate tissue from A) Sedentary PCa rats compared with sedentary CONT rats and B) Exercised PCa rats compared with sedentary PCa rats. Selected the top 10 terms tested with Fisher exact test and false discovery rate (FDR) testing with a *p*-value < 0.05

To enhance the understanding of the biological role of the differentially expressed and phosphorylated proteins in the context of this study, the lists of differentially proteins modulated by PCa and exercise were subjected to enrichment analyses to determine overrepresented protein classes, biological processes, molecular functions, and cellular components (Fig. 5, Supplementary Table 1 and Supplementary Table 2). The 15 proteins regulated by PCa were assigned into a major PANTHER Protein Classes: “non-receptor serine/threonine protein kinase” (Fig. 5A). The most enriched GO biological processes were associated with cell signalling and cellular response to stress. A selection basis for signalling proteins is intrinsic for the antibody microarray that we used, although it covered a wide range of signalling pathways. Regarding GO molecular functions, we found an enrichment of ontologies related to protein binding and kinase activity. No GO cellular components with statistical significance were found. The 25 proteins modulated by exercise in the set of PCa were assigned into a major PANTHER Protein Class: “non-receptor serine/threonine protein kinase” (Fig. 5B). The most enriched GO biological processes were associated with regulation of cell differentiation and cell structure organization. Regarding GO molecular functions, as expected by the methodology, an enrichment of ontologies related to kinase activity and protein binding was observed. The GO cellular components most overrepresented were protein complexes and cell-associated structures.

**Fig. 6:**
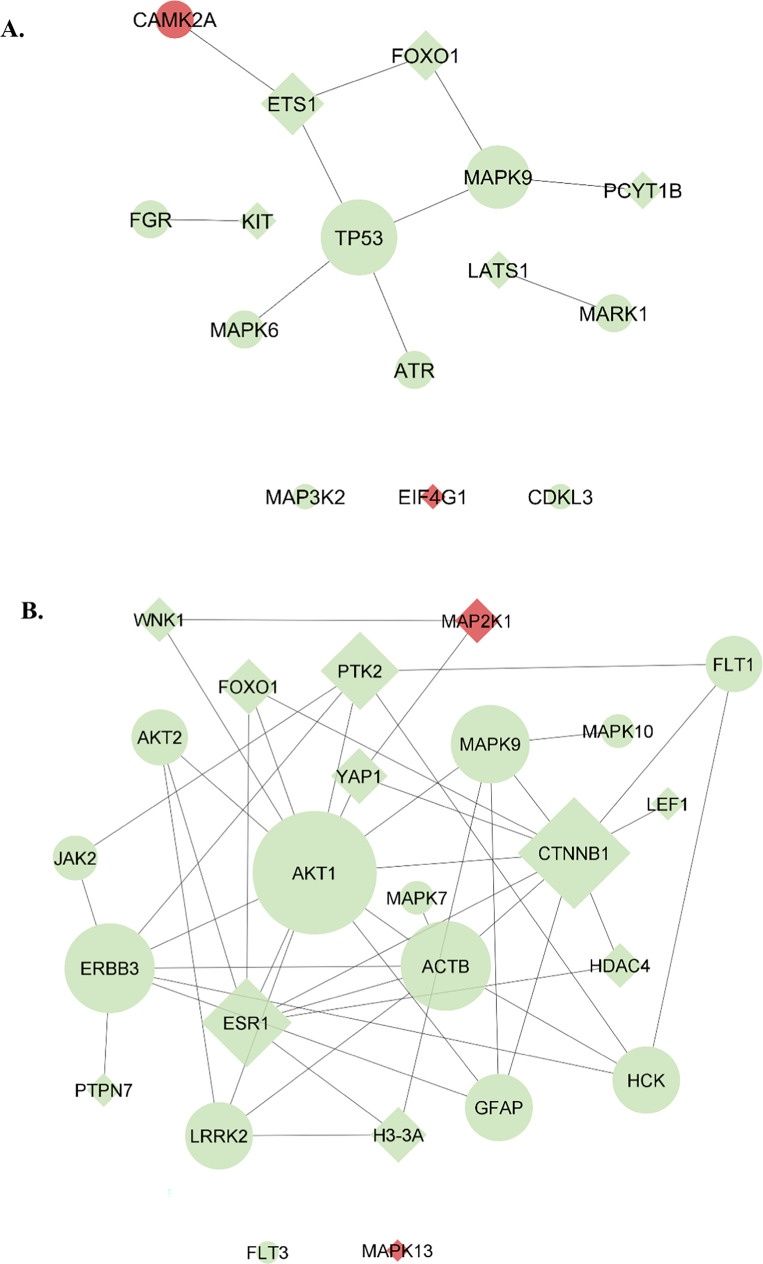
Protein–protein interaction network involving (A) the 15 proteins identified as differentially expressed or phosphorylated in sedentary PCa rats compared with sedentary CONT rats and (B) the 25 proteins identified as differentially expressed or phosphorylated between exercised PCa rats compared with sedentary PCa rats. Nodes’ colour represents the expression alteration (green: upregulated; red: downregulated); nodes’ shape indicates if the alteration was found for pan-specific form (circles). phosphorylation form (diamond)

Protein–protein interaction networks were constructed to identify potential relationships among the proteins found to be differentially regulated in prostate tissue from SED + PCa rats and paired tissue from CONT + SED rats (Fig. 6A), and also in prostate tissue from EX + PCa rats compared with SED + PCa rats (Fig. 6B). The network resulting from the differentially proteins modulated by PCa has identified TP53, MAPK9 and ETS1 as the most central nodes with greatest closeness. The protein–protein interaction network that was constructed with the proteins differentially regulated in the prostate from EX + PCa rats when compared with SED + PCa animals showed 25 nodes highly interconnected, with AKT1 and CTNNB1 as the most central ones for having more interactors (detailed analysis of the protein–protein interaction networks is presented as Supplementary information).

## Discussion

In the current study, we aimed to understand how lifelong exercise training promotes the remodelling of the immune system and prostate signalome in a rat model of PCa. For this purpose, a rat model of PCa that reflects man prostate carcinogenesis was used [14, 15]. Male rats were subject to moderate intensity treadmill exercise training for a long period of time—53 weeks—which in man corresponds to approximately 40 years of exercise, according to the calculations performed by Pallav Sengupta [34]. It is worth to note that, to the best of our knowledge, this study was the first one to evaluate the effects of lifelong exercise training on prostate carcinogenesis after carcinogen exposure, using an integrative approach. In our study, exercise training decreased animal body weight but increased prostate weight to tibia length ratio in EX + CONT and EX + PCa, when compared to respective sedentary groups. Besides, the same pattern was observed when comparing PCa groups with respective controls. Our results are in accordance with previous works in which PCa induction protocol and exercise training decreased animals’ body weight but increased prostate weight [35–39]. Although these PCa induction protocol is based on testosterone implants, our results suggested that the increase of testosterone serum values can be due to exercise, because control exercised animals (non-exposed to testosterone) also have a high testosterone concentration (not statistically significant). Indeed, exercise training is known to change the circulating levels of sex hormones, such as testosterone and estradiol, as well as their transporter protein (hormone-binding globulin, SHBG) and their receptors expression [40–43]. However, there is no consensus concerning to the trend of such alterations since both a decrease and an increase of SHBG levels were described. Consequently, the influence of exercise in modulating these relations is not clearly understood. Moreover, the contraction of skeletal muscles during exercise and the metabolic alterations in adipose tissue can raise circulating testosterone levels [4, 44–48]. “We observed prostate inflammation, acute and chronic, in all groups: sedentary and exercised. These patterns of inflammation were focal and not diffuse. In our opinion, age is associated with prostate inflammation and exercise did not revert this finding. Concerning to PCa induced animals, beyond the age, the induction protocol is associated with the development of local inflammation, which is in accordance with published studies [49, 50], and not reversed by our exercise program. However, in our study, PCa was not associated with systemic inflammation as shown by CRP and TWEAK serum results. The high circulating levels of testosterone, already described, may have interfered with the adaptations promoted by exercise, like the anti-inflammatory effect of aerobic exercise in cancer [51].

Concerning to the circulating lymphocytes, exercise promoted the increase of CD161^+^ cells in PCa rats (*p* = 0.058), a marker of NK cells. These results are in agreement with those previously described in the literature, concerning the beneficial effects of exercise by increasing NK cells, a cell population with anti-tumour immunity [52, 53]. The same pattern was observed with γδ T cells. These cells are reported to have several advantages, they migrate to peripheral tissues rather than to lymphoid organs, and they are independent of major histocompatibility complex-dependent antigen [54]. Thus, γδ T cells are considered anti-tumour, cytotoxic lymphocytes and may represent good alternative targets for immunotherapy [55]. According to our results, we observed a significant increase of γδ T cells in exercised PCa rats compared to sedentary PCa rats, corroborating the beneficial effects of exercise in the modulation of immune system during PCa induction [7, 56]. Looking at the same lymphocyte immunohistochemical markers on dorsolateral prostate tissue, a similar pattern was identified: a decrease in CD4^+^/ CD8^+^ ratio, which indicate a greater availability of T cells with cytotoxic capacity (cells with antitumour response). This is in accordance with literature and reinforce the beneficial effect of exercise in modulating the immune system through the mobilization and redistribution of effector lymphocytes in prostate tissue, associated with an antitumor response [7, 57, 58]. A trend to increased infiltration of CD68 + cells was observed in exercised rats (EX + CONT and EX + PCa) compared to respective sedentary rats (SED + CONT and SED + PCa). No differences were seen in the infiltrating tumour-support macrophages (M2), represented by CD163 + , cells. The M2 cells may inhibit the cytotoxic and inflammatory functions of M1 macrophages, promoting tumour progression [59, 60]. Published data has indicated that exercise training may increase the levels of macrophages [61].

The spectrum of dorsolateral prostate histopathological lesions identified in this work are like those described in man prostate. Although control animals also developed prostate lesions, the number of lesions was lower than in PCa groups (*p* < 0.05). The occurrence of these lesions in non-exposed animals can be explained by ageing, as reported in man [62]. The percentage and spectrum of prostate lesions identified was similar between PCa sedentary and PCa exercised groups. So, the exercise did not interfere in the development of dorsolateral prostate lesions. Different results were described in the literature, although with other type of PCa models and exercise programs [63–65]. These discrepant results reinforce the importance of continuing to study this issue.

The prostate lesions had a low proliferation rate in both sedentary and exercised induced groups, with no clear effect of exercise in this parameter. Similarly, Malicka et al*.* (2015) [66] who used a chemically-induced mammary cancer model submitted to treadmill exercise for 12 weeks, did not observe differences in Ki-67 expression. In a general way, our results showed a high immunopositivity to AR and low positivity to ERα expression in dorsolateral prostate tissue. AR is expressed in prostatic epithelial and stromal cells, and mediates the response to androgens [67]. In PCa, mutation or amplification of *AR* gene increase sensitivity of neoplastic cells to androgen and other steroid hormones [68, 69]. Thus, our data suggest that prostate cells developed resistance to testosterone, similarly to that reported for testosterone replacement therapy in men [70]. Indeed, in our protocol the rats were exposed to exogenous testosterone during approximately 10 months. High doses of testosterone in circulation are expected to boost the increase of estrogen levels through peripheral aromatase conversion of testosterone, which may happen at prostate stroma. Aromatization to 17β-oestradiol has been hypothesized to be important in testosterone-stimulated prostate growth [71]. Estrogens/ER signalling plays an important role in the growth and differentiation of normal prostate tissue and also in prostate carcinogenesis [72–74]. Prostate tissue expresses ERα and ERβ. The latter is mainly found in basal cells and has a predominantly protective effect against PCa development, mediating the antiproliferative, anti-inflammatory and anti-carcinogenic effects of estrogen [73, 75–78]. ERα is found mainly in stromal cells and has an oncogenic role. It has been suggested that the presence and activity of ERα is required for aberrant proliferation, inflammation and cancer in human prostate [67, 73, 75, 79]. However, contradictory findings on the role of these receptors in the PCa continue to emerge [80–84]. Taking into consideration our results, immunohistochemical analysis of dorsolateral prostate sections did not support the role of ERα in prostate remodeling, once low expression was detected and was not influenced by PCa and/or exercise training. However, proteome profiling of prostate tissue with an antibody microarray highlighted the effect of exercise on the content of the phosphorylated (at Ser-104) form of this receptor (coded by the gene *ESR1*). Ser-104 was reported to be targeted by mTOR in breast cancer and to stimulate estrogen/ERα-mediated gene expression [85]. Our data support the interaction of Akt, but not of mTOR, with ERα, being probably the kinase involved in this receptor activation promoted by exercise in prostate cells. ERα signaling was reported to stimulate prostate growth by increasing phosphorylated Akt and its effectors, including CDK1 and p27 [86]. Thus, more than impacting the levels of total ERα, exercise seems to increase ERα phosphorylation and activity.

FOXO1 is also a target of Akt, according to our and previous data [87], and was implicated in prostate cell proliferation, differentiation and apoptosis through the regulation of multiple genes. Once phosphorylated by Akt, its transcriptional activity is inhibited. Thus, our data indicate that exercise inhibits the transcription of genes under FOXO1 regulation. This data is also supported by exercise-related decreased phosphorylation of MAPK13, which is the delta isoform of the p38 MAP kinase family and it is involved in epithelial PCa cells differentiation [88]. Another signaling pathway modulated in prostate by lifelong exercise involves the catenin-1 or β-catenin (coded by the gene *CTNNB1*). Aberrant Wnt/β-catenin signaling has been linked to several human cancers [89, 90] including PCa [87, 91, 92]. β-catenin may directly interact with ERα, suggesting that these proteins may act synergistically to regulate gene transcription. Phosphorylation of β-catenin by Akt was previously associated to the activation of β-catenin/LEF pathway and the proliferation of the prostate cell line PC-3 [93]. Thus, exercise seems to regulate ERα/β-catenin/LEF signaling in prostate tissue, which impact on tumorigenic processes needs to be further explored in PCa setting. Still, increased phosphorylation (Tyr-397) and consequent activation of FAK (coded by PTK2 gene) has been associated with elevated invasion and metastatic potential in PCa [94]; however, no histological signs of increased malignancy were observed in trained PCa rats. FAK phosphorylation may be triggered by VEGF indirectly and stimulate cell motility, as reported in an in vitro model [95]. All groups showed high immunopositivity for VEGF (> 75% of immunopositive cells extension) and exercised PCa group showed an increase when compared with PCa sedentary group, though not statistically significant. Moreover, proteome data showed an increase of VEGFR1 (coded by *FLT1* gene) phosphorylation in the prostate from trained PCa rats, and its activation seems to have more than an angiogenic role in this tissue [95]. Taken together, enrichment in signalling pathways of the differentially expressed proteins highlights the overrepresented pathways modulated by exercise in prostate carcinogenesis, such as endocrine resistance, PI3K-AKT, FOXO, MAPK pathways, which may explain the greater weight of the prostate of trained PCa rats, compared with sedentary PCa animals.

“Some limitations can be identified in our work. It seems that the induction of prostate macroscopic lesions in male rats is difficult, because 11 months after PCa induction, that correspond to 15 months of rats’ age, these lesions were not observed. Longer protocols do not seem appropriate to us, because the animals’ mortality will increase with their aging, and the acquisition of samples can be compromised. Future studies should evaluate other types of exercise (e.g. anaerobic, strength), intensity (low, moderate or high) and duration of exercise sessions (short or long training). According to our results, it is important to know the effects that different exercise programs may have in PCa development and evolution, to establish the most appropriate plan for maximum benefits.

## Conclusion

Our data highlight the exercise-induced remodelling of peripheral lymphocyte subpopulations and lymphocyte infiltration in prostate tissue, characterized by CD4^+^, CD8^+^ and CD68^+^ cells, which reinforce the anti-tumour role of exercise. Proteomic data confirms the great impact of sex hormones on prostate carcinogenesis, exacerbated by lifelong exercise training. Indeed, endocrine resistance, PI3K-AKT, FOXO, MAPK pathways were the overexpressed pathways modulated by exercise training in this PCa model. These signalling pathways are associated with cell proliferation and tumour development; however, NK, γδ T cells and, eventually, other mechanisms seem to have acted to slow down prostate carcinogenesis, once no signs of increased malignancy were seen in trained PCa rats. The exercise had no impact on ERα and AR expression, although phosphorylation of ERα at the stimulatory Ser-104 site was enhanced. Our data also suggest that the duration and type of endurance exercise implemented did not avert the effect of chronic exposure to testosterone in prostate remodelling.

These data deserve more investigation using other exercise protocols, such as anaerobic exercises or active days and rest days interspersed, to clarify the effect of exercise training on PCa in this animal model.

### Supplementary Information

Below is the link to the electronic supplementary material.

Supplementary file1 (DOCX 123 kb)

## Data Availability

Data and resource sharing are available upon request.
