# Supplementary material for: Lifelong exercise training promotes the remodelling of the immune system and prostate signalome in a rat model of prostate carcinogenesis
Source: GeroScience. 2023 May 12;46(1):817–40. doi: 10.1007/s11357-023-00806-5 (PMC10828357; doi:10.1007/s11357-023-00806-5)
Supplement: Supplementary file 1 — Supplementary file1 (DOCX 123 kb) [file 11357_2023_806_MOESM1_ESM.docx]

**Supplementary material**


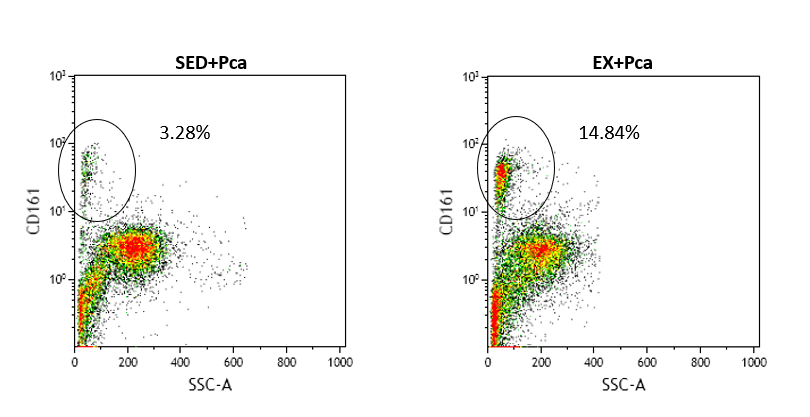


**Supplementary Figure 1** - Representative flow cytometry plots of natural killer (NK) cell population (CD161+ cells) comparing induced sedentary (SED+Pca) and induced exercised (EX+Pca) animal case.

| **Supplementary Table 1 -** Identification of proteins from each overrepresented PANTHER protein classes and GO ontology categories of prostate tumour tissue PCa sedentary rats compared with sedentary control rats. Selected the top 10 terms tested with Fisher exact test and false discovery rate testing with a p-value <0.05. | | |
| --- | --- | --- |
| **PANTHER protein classes** | **Up-regulated** | **Down-regulated** |
| non-receptor serine/threonine protein kinase (PC00167) | CDKL3; MARK1; MAPK9; MAPK6; ATR; LATS1 | CAMK2A |
| **GO biological processes** | **Up-regulated** | **Down-regulated** |
| positive regulation of cardiac muscle cell apoptotic process (GO:0010666) | TP53 | CAMK2A |
| replicative senescence (GO:0090399) | TP53; TATR |  |
| regulation of mitochondrial membrane permeability involved in apoptotic process (GO:1902108) | TP53 | CAMK2A |
| positive regulation of gene silencing by miRNA (GO:2000637) | TP53 | EIF4G1 |
| cellular response to gamma radiation (GO:0071480) | TP53; ATR |  |
| peptidyl-serine phosphorylation (GO:0018105) | MARK1; MAPK9; ATR: LATS1 | CAMK2A |
| cellular response to reactive oxygen species (GO:0034614) | MAPK9; FOZO1; ETS1 |  |
| protein autophosphorylation (GO:0046777) | ATR; FGR; KIT | CAMK2A |
| positive regulation of protein-containing complex assembly (GO:0031334) | MAPK9; TP53; ATR | EIF4G1 |
| MAPK cascade (GO:0000165) | MAPK9; MAPK6; AP3K2; KIT | CAMK2A |
| cellular response to external stimulus (GO:0071496) | FOXO1; TP53; MAP3K2 | EIF4G1 |
| regulation of cellular response to stress (GO:0080135) | FOXO1; TP53; ATR | CAMK2A; EIF4G1 |
| **GO molecular function** | **Up-regulated** | **Down-regulated** |
| MAP kinase activity (GO:0004707) | MAPK9; MAPK6 |  |
| histone acetyltransferase binding (GO:0035035) | TP53; ETS1 |  |
| protein phosphatase 2A binding (GO:0051721) | FOXO1; TP53 |  |
| ATP binding (GO:0005524) | CDKL2; MARK1; MAPK9; TP53; MAPK6; ATR; FGR; MAP3K2; LATS1; KIT | CAMK2A; EIF4G1 |
| transcription factor binding (GO:0008134) | MAPK9; FOXO1; TP53; ETS1; LATS1 |  |
| **GO cellular component** | **Up-regulated** | **Down-regulated** |
| Nonstatistical meaning |  |  |

| **Supplementary Table 2** - Identification of proteins from each overrepresented PANTHER protein classes and GO ontology categories in prostate tissue from PCa exercised rats compared with PCa sedentary rats. Selected the top 10 terms tested with Fisher exact test and false discovery rate testing with a p-value <0.05. | | |
| --- | --- | --- |
| **PANTHER protein classes** | **Up-regulated** | **Down-regulated** |
| non-receptor serine/threonine protein kinase (PC00167) | MAPK7; MAPK10; WNK1; AKT2; AKT1; MAPK9 | MAP2K1 |
| **GO biological processes** | **Up-regulated** | **Down-regulated** |
| JUN phosphorylation (GO:0007258) | MAPK9; MAPK10 |  |
| negative regulation of long-chain fatty acid import across plasma membrane (GO:0010748) | AKT1; AKT2 |  |
| trachea formation (GO:0060440) | CTNNB1 | MAP2K1 |
| detection of muscle stretch (GO:0035995) | CTNNB1; PTK2 |  |
| peripheral nervous system myelin maintenance (GO:0032287) | AKT1; AKT2 |  |
| negative regulation of heterotypic cell-cell adhesion (GO:0034115) | MAPK7; WNK1 |  |
| Bergmann glial cell differentiation (GO:0060020) | GFAP | MAP2K1 |
| regulation of nephron tubule epithelial cell differentiation (GO:0072182) | YAP1; CTNNB1 |  |
| positive regulation of mitochondrial membrane potential (GO:0010918) | AKT1; AKT2 |  |
| adherens junction assembly (GO:0034333) | ACTB; CTNNB1 |  |
| **GO molecular function** | **Up-regulated** | **Down-regulated** |
| JUN kinase activity (GO:0004705) | MAPK9; MAPK10 |  |
| estrogen receptor activity (GO:0030284) | ESR1; LEF1 |  |
| nitric-oxide synthase regulator activity (GO:0030235) | AKT; ESR1 |  |
| vascular endothelial growth factor-activated receptor activity (GO:0005021) | FLT1; FLT3 |  |
| MAP kinase kinase activity (GO:0004708) | MAPK10; LRRK2 | MAP2K1 |
| transcription coactivator binding (GO:0001223) | FOXO1; CTNNB1; ESR1; LEF1 |  |
| estrogen receptor binding (GO:0030331) | CTNNB1; ESR1; LEF1 |  |
| non-membrane spanning protein tyrosine kinase activity (GO:0004715) | JAK2; PTK2; HCK |  |
| beta-catenin binding (GO:0008013) | FOXO1; CTNNB1; ESR1; LEF1 |  |
| protein kinase activator activity (GO:0030295) | ERBB3; WNK1 | MAP2K1 |
| **GO cellular component** | **Up-regulated** | **Down-regulated** |
| Wnt signalosome (GO:1990909) | CTNNB1; LEF1 |  |
| beta-catenin-TCF complex (GO:1990907) | CTNNB1; LRRK2 |  |
| caveola (GO:0005901) | JAK2; CTNNB1; LRRK3; Hck |  |
| protein-DNA complex (GO:0032993) | CTNNB1; H3F3B; ESR1; LEF1 |  |
| lamellipodium (GO:0030027) | ACTB; AKT1; CTNNB1; PTK2 |  |
| focal adhesion (GO:0005925) | JAK2; ACTB; CTNNB1; PTK2; FLT1; HCK | MAP2K1 |
| actin cytoskeleton (GO:0015629) | HDAC4; ACTB; PTK2; FLT1; HCK |  |
| postsynapse (GO:0098794) | JAK2; ACTB; AKT1; CTNNB1; LRRK2; PTK2 |  |
| nucleoplasm (GO:0005654) | JAK2; HDAC4; YAP1; MAPK7; ACTB; MAPK9; MAPK10; AKT1 |  |
| cytosol (GO:0005829) | FLT3; GFAP; HCK; PTPN7; LRRK2; MAPK7; MAPK10; ACTB; WNK1; ATK2  PTK2; MAP2K1; FOXO1; CTNNB1; AKT1; MAPK9; MAPK13; HDAC4; JAK2; ESR1; YAP1 | MAP2K1; MAPK13 |

**Supplemental information**

**Protein- protein interaction network**

The network resulting from the differentially proteins modulated by PCa has an average number of neighbours of 2.000 and three isolated nodes (MAP3K2, EIF4G1 and CDKL3). TP53, MAPK9 and ETS1 were identified as the most central nodes for having highest degrees (4, 3 and 3, respectively), as well as exhibiting the greatest closeness (0.700, 0.583 and 0.583, respectively) and betweenness centrality (0.619, 0.357 and 0.357). Closeness centrality indicates how close a node is to all other nodes in the network and thus, lower values indicate more central nodes. Regarding betweenness centrality, it is a measure of the fraction of shortest paths passing through a vertex, so higher numbers indicate that the node is more central to the network. The protein-protein interaction network that was constructed with the proteins differentially regulated in the prostate from EX+PCa compared with SED+PCa showed 25 nodes highly interconnected, with an average number of neighbours of 4.000 and with two isolated nodes (FLT3 and MAPK13). AKT1 and CTNNB1 were identified as the most central nodes for having the highest degrees (12 and 10, respectively), as well as exhibiting the greatest closeness centrality (0.688 and 0.611, respectively), and betweenness centrality (0.385 and 0.252, respectively). Moreover, the mean clustering coefficient of this network was C=0.34. The clustering coefficient is a measure of how nearest neighbouring nodes of a node are connected to each other. When the nodes are tightly connected to each other the clustering coefficient is near 1. If the network is a sparse random uncorrelated network of finite size N, the clustering coefficient expected is close to zero.

The enrichment in signalling pathways of the differentially expressed proteins in the prostate of SED+PCa rats and SED+CONT rats was investigated using data from KEGG database. Six genes were not included in KEGG output: *FGR*, *CDKL3*, *EIF4G1*, *MARK1*, *MAPK6*, and *PCYT1B*. After applying Benjamini correction, we did not found results with statistical significance (*p*>0.05). Besides, in the comparison of EX+PCa rats and SED+PCa rats using the KEGG database, 42.3% of the proteins found were already annotated to the KEGG “Pathways in Cancer” class (F.E. = 7.1, *p*=8.0E-6): AKT1, AKT2, JAK2, CTNNB1, ESR1, FLT3, FOXO1, MAPK10, MAPK9, MAP2K1, and PTK2. Two genes were not included in KEGG output: *WNK1* and *Lef1*. Moreover, five proteins (19%) were specifically annotated to the KEGG “Prostate Cancer” class (F.E. = 17.6, *p*=5.0E-4): AKT1, AKT2, CTNNB1, FOXO1, and MAP2K1. Overrepresented pathways also included MAPK signalling pathway (F.E. = 12.8, *p*=1.3E-7), Focal adhesion (F.E. = 15.3, *p*=9.0E-7), Proteoglycans in cancer (F.E. = 15.0, *p??* = 9.3E-7), Prolactin signalling pathway (F.E. = 39.0, *p*=9.5E-8), Endocrine resistance (F.E. = 27.9, *p*=2.6E-7), Growth hormone synthesis, secretion and action (F.E. = 23.0, *p*=6.8E-7), PI3K-Akt signalling pathway (F.E. = 7.7, *p*=1.6E-4), ErbB signalling pathway (F.E. = 28.1, *p*=1.5E-6), FoxO signalling pathway (F.E. = 18.2 , *p*=1.2E-5), Rap1 signalling pathway (F.E. = 11.4, *p*=1.1E-4) and Ras signalling pathway (F.E. = 10.3, *p*=1.5E-4).
